# Enrichment of the embryonic stem cell reprogramming factors Oct4, Nanog, Myc, and Sox2 in benign and malignant vascular tumors

**DOI:** 10.1186/s12907-015-0018-0

**Published:** 2015-09-26

**Authors:** Clarissa N. Amaya, Brad A. Bryan

**Affiliations:** Department of Biomedical Sciences, Paul L. Foster School of Medicine, Texas Tech University Health Sciences Center, El Paso, TX USA

## Abstract

**Background:**

The “stem cell theory of cancer” states that a subpopulation of cells with stem cell-like properties plays a central role in the formation, sustainment, spread, and drug resistant characteristics of malignant tumors. Recent studies have isolated distinct cell populations from infantile hemangiomas that display properties equivalent to aberrant progenitor cells, suggesting that, in addition to malignant tumors, benign tumors may also contain a stem cell-like component.

**Methods:**

In this study, the expression levels of the embryonic stem cell reprogramming factors Oct4, Nanog, Myc, Sox2, and Klf4 were examined via immunohistochemistry in a panel of 71 benign, borderline, and malignant vascular tumors including capillary hemangioma, cavernous hemangioma, granulomatous hemangioma, venous hemangioma, hemangioendothelioma, hemangiopericytoma, and angiosarcoma. Antigenicity for each protein was quantified based on staining intensity and percentage of tissue positive for each antigen, and subsequently compared to data obtained from two control tissue sets: 10 vascular tissues and a panel of 58 various malignant sarcomas.

**Results and discussion:**

With the exception of Myc (which was only present in a subset of benign, borderline, and malignant tumors), Oct4, Nanog, Sox2, and Klf4 were detectable at variable levels across both normal and diseased tissues. Semi-quantitative evaluation of our immunohistochemical staining revealed that protein expression of Oct4, Nanog, Myc, and Sox2, but not Klf4, was significantly increased in benign, borderline, and malignant vascular tumors relative to non-diseased vascular tissue controls. Interestingly, the enhanced levels of Oct4, Nanog, Myc, and Sox2 protein were approximately equivalent between benign, borderline, and malignant vascular tumors.

**Conclusions:**

These findings provide supporting evidence that enrichment for proteins involved in pluripotency is not restricted solely to malignant tumors as is suggested by the “stem cell theory of cancer”, but additionally extends to common benign vascular tumors such as hemangiomas.

**Electronic supplementary material:**

The online version of this article (doi:10.1186/s12907-015-0018-0) contains supplementary material, which is available to authorized users.

## Background

The origin of cancer remains unclear, however the “cancer stem cell theory” postulates that a subpopulation of cancer cells with stem cell-like properties is responsible for sustaining long term tumor growth [1]. In addition, cancer stem cells give rise to metastases and can act as a reservoir that potentially leads to relapse after treatment has eliminated all observable signs of the cancer. These cancer stem cells are believed to be genotypically and/or phenotypically related to normal stem cells and share many of the features of normal stem cells such as self-renewal, drug resistance, and a proliferative potential to generate a multi-potent cellular lineage [2, 3]. The core transcription factors that control “stemness” in embryonic stem cells include Oct4, Sox2, Nanog, Myc, and Klf4, and the combination of these factors has been shown to successfully reprogram differentiated somatic cells into pluripotent stem cells [4]. There is substantial evidence that cancer stem cells express these specific markers and their activity contributes to the oncogenic properties inherent in this disease [5].

In addition to malignant tumors, benign prostate, breast, and angiomyolipoma tumors express various stem cell markers, suggesting the expression of these markers is not limited exclusively to malignant tumors [6–9]. It was recently reported that benign infantile hemangiomas, which are the most common tumors of infancy, express higher levels of neural crest and stem cell markers at the mRNA level than dermal microvascular endothelial cells [10], and within this tumor type resides multiple cellular subpopulations expressing Oct4 and Nanog proteins [11]. Moreover, it was recently revealed that a clonogenic subpopulation of cells isolated from cutaneous infantile hemangiomas was capable of differentiating into endothelial cells, smooth muscle, or adipocytes [12], suggesting that a stem cell-like component may drive the etiology of this benign vascular tumor. These fascinating findings suggest that the “stem-cell theory of cancer” may serve as a more generalized theory than is currently accepted, and extend to benign vascular tumors.

Thus, in this study we used immunohistochemical analysis to examine the expression of the stem cell reprogramming factors Oct4, Sox2, Nanog, Myc, and Klf4 in 71 diverse benign and malignant vascular tumors. Our findings surprisingly revealed that, relative to normal endothelial tissues, staining of benign and malignant vascular tumors demonstrated significantly higher expression of these stem cell reprogramming factors.

## Methods

### Immunohistochemistry (IHC)

Blood vessel disease spectrum tissue arrays containing various vascular tumors and non-diseased controls were purchased from US Biomax, Inc. (#SO8010). The sarcoma tissue arrays were purchased from Novus Biologicals (#NBP2 = 30332). For detection of protein expression, tissue arrays were labeled with anti-Myc (Cat# ab32072; Abcam), anti-Oct4 (Cat# ab18976; Abcam), anti-Sox2 (Cat# ab97959; Abcam), anti-Klf4 (Cat# ab118961; Abcam), and anti-Nanog (Cat# ab80892; Abcam) antibodies. Antigenicity was detected using Alkaline Phosphatase reactivity (CellMarque). Positive (primary antibody included) and negative (primary antibody excluded) controls from human intestine (Klf4), human testicle (Oct4 and Nanog), rat brain (Sox2), or human colon cancer (Myc) which have been reported by the Human Protein Atlas (HPA) (www.proteinatlas.org) were subjected to immunohistochemistry to validate the specificity of each antibody tested (Additional file 1: Figure S1). In addition, immunohistochemistry for each antigen was performed on adipose tissue as a negative control, given the HPA revealed no to very low expression of each protein in this tissue type (Additional file 1: Figure S1). Immunopositivity was quantified by two metrics: the percentage of tissue with positive staining (<25 %, 25–50 %, 50–75 %, or >75 %) and the staining intensity (0 = no staining, + = weak staining, ++ = moderate staining, +++ = high staining). IHC scores were determined by multiplying the staining intensity (0 = 0, + = 1, ++ = 2, +++ = 3) by the percent of tissue stained (<25 % = 1, 25–50 % = 2, 50–75 % = 3, >75 % = 4) based on previously described methods [13]. For statistical analysis, the Mann-Whitney rank sum test was used. Statistical significance was determined if the two-sided P value of the test was < 0.05. Use of human tissues for research was approved by TTUHSC board review #11027.

## Results

Included in this study were 71 diseased vascular tissue samples originally collected from human patients, representing malignant (seven angiosarcomas, two hemangiopericytomas), borderline (six hemangioendothelioma), and benign (five infantile hemangioma, one capillary hemangioma, 45 cavernous hemangiomas, three granulomatous hemangiomas, one venous hemangioma) vascular tumors and one thrombophlebitis. Known characteristics of patients grouped according to biopsy classification are reported in Table 1. As controls, we included two tissue sets in this analysis: 1) ten non-diseased blood vessel tissues and 2) a diverse panel of 58 human sarcoma tumors. The non-diseased blood vessel tissues were chosen to evaluate the expression of stem cell reprogramming factors in normal vasculature, while the various sarcomas were selected to compare the levels of stem cell reprogramming factors in borderline and malignant vascular sarcomas to that of other malignant mesenchymal tumors.

**Table 1:** Vascular tumor and control patient characteristics

Variable	Overall	Malignant	Borderline	Benign	Normal
# patient samples	81	9	6	56	10
Age [mean years (s.d.)]	41 ± 17	53 ± 19	36 ± 15	40 ± 17	34 ± 14
Age [median years (range)]	42 (80)	53 (64)	35 (44)	42 (71)	32 (44)
Sex	42 F, 39 M	4 F, 5 M	6 F, 0 M	27 F, 29 M	5 F, 5 M

**Fig. 1:**
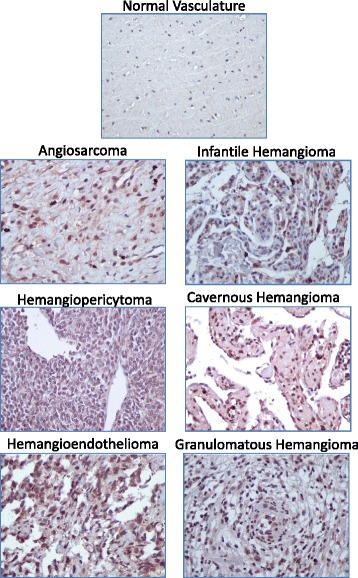
Representative images of Oct4 staining in normal and vascular tumor tissues. Immunopositivity for Oct4 protein is represented by brown staining. Positive control *(left panel)* = human testicle; negative control *(right panel)* = human testicle with no added primary antibody. 400× total magnification for each image

**Fig. 2:**
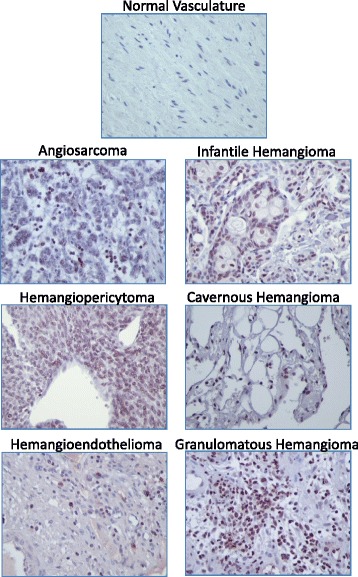
Representative images of Nanog staining in normal and vascular tumor tissues. Immunopositivity for Nanog protein is represented by brown staining. Positive control *(left panel)* = human testicle; negative control *(right panel)* = human testicle with no added primary antibody. 400× total magnification for each image

**Fig. 3:**
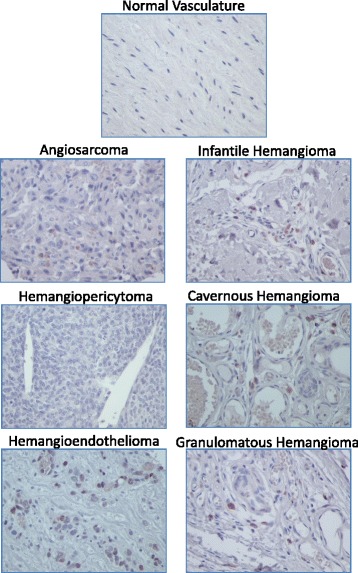
Representative images of Myc staining in normal and vascular tumor tissues. Immunopositivity for Myc protein is represented by brown staining. Positive control *(left panel)* = human colon cancer; negative control *(right panel)* = human colon cancer with no added primary antibody. 400× total magnification for each image

**Fig. 4:**
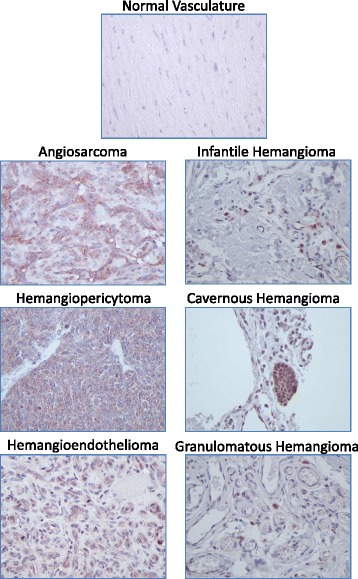
Representative images of Sox2 staining in normal and vascular tumor tissues. Immunopositivity for Sox2 protein is represented by brown staining. Positive control *(left panel)* = rat brain; negative control *(right panel)* = rat brain with no added primary antibody. 400× total magnification for each image

**Fig. 5:**
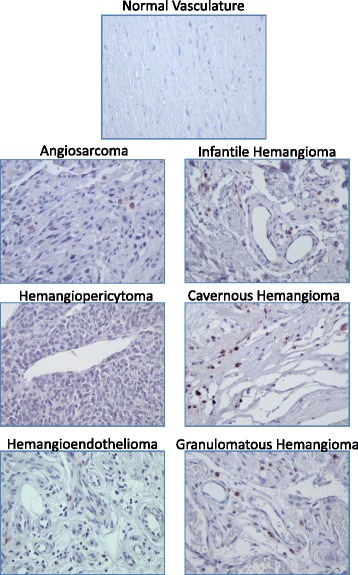
Representastive images of Klf4 staining in normal and vascular tumor tissues. Immunopositivity for Klf4 protein is represented by brown staining. Positive control *(left panel)* = human intestine; negative control *(right panel)* = human intestine with no added primary antibody. 400× total magnification for each image

**Fig. 6:**
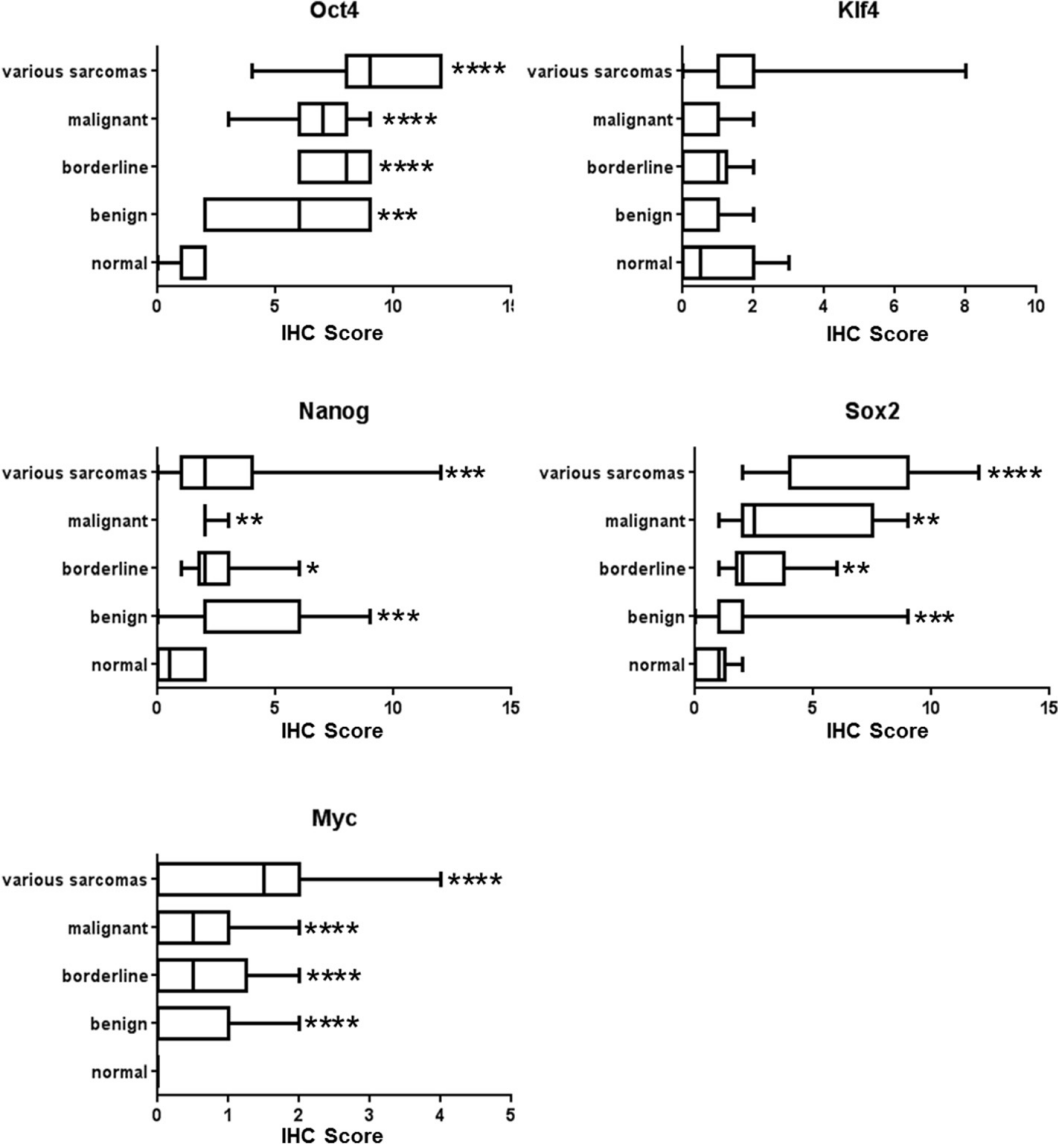
Antigenicity for embryonic stem cell reprogramming factors in normal tissue and vascular tumors. Box and whisker plots depicting the IHC scores for Oct4, Nanog, Myc, Sox2, and Klf4 in normal vasculature, benign, borderline or malignant vascular tumors, or across a panel of various sarcomas. The Mann-Whitney rank sum test was used to determine statistical significance. Significance was determined if the two-sided P value of the test was < 0.05. Asterisks indicate level of significance relative to normal vasculature (* *p* < 0.05, ** *p* < 0.05, *** *p* < 0.005, **** *p* < 0.0005)

IHC staining for the stem cell reprogramming factors Oct4, Nanog, Myc, Klf4, and Sox2 was performed in the vascular tumor samples as well as the two control tissue sets. Representative images of each staining are depicted in Figs. 1, 2, 3, 4 and 5. With the exception of Myc, each of these proteins was detectable at variable levels across non-diseased vascular tissues, ranging from 50 % of normal tissues displaying Nanog and Klf4 immunoreactivity to 90 % of normal tissues displaying Oct4 immunoreactivity (Table 2). Expression of these “stem cell regulators” in non-diseased adult tissue is not surprising given that the HPA reports detection of Oct4, Nanog, Klf4, and Sox2 in approximately 70, 11, 29, and 51 % of normal human tissues, respectively. Though HPA reports Myc expression in 56 % of normal human tissues, we did not detect this protein in any non-diseased vascular tissues tested in this analysis. While immunostaining for these stem cell regulators was observed in non-diseased vasculature, the IHC score for these tissues was relatively low given that staining intensity for each stem cell marker was weak to moderate and often occurred in a very small fraction (<25 %) of the cells comprising the tissue (Fig. 6, Additional file 2: Table S1).

**Table 2:** Percentage of tumors with positive antigenicity for embryonic stem cell reprogramming factors

Protein	Normal	Benign	Borderline	Malignant	Various Sarcomas
Oct4	90 %	100 %	100 %	100 %	100 %
Nanog	50 %	98 %	100 %	100 %	100 %
Myc	0 %	46 %	50 %	50 %	72 %
Sox2	60 %	98 %	100 %	100 %	100 %
Klf4	50 %	59 %	67 %	63 %	72 %

In contrast to the non-diseased vascular rich tissue controls, benign vascular tumors and the single thrombophlebitis sample exhibited significantly increased staining (in both intensity and percentage of positive tissue) for Oct4, Nanog, Myc, and Sox2, with no statistically significant increase in antigenicity for Klf4 (Fig. 6, Additional file 2: Table S1). It is worth noting that unlike the absence of Myc expression in normal vasculature, 46 % of the benign tumors tested were positive for Myc protein. The data obtained from malignant and borderline vascular tumors were remarkably similar to that demonstrated from the benign vascular tumors. Malignant and borderline vascular sarcomas displayed 100 % immunoreactivity for Oct4, Nanog, and Sox2, while Myc protein was present in 50 % of malignant and borderline vascular tumors (Table 2). The IHC scores for all proteins tested except Klf4 were significantly increased in the malignant and borderline vascular tumors relative to the non-diseased controls, and were surprisingly very similar to the levels observed in benign vascular tumors. The elevated IHC scores observed for malignant and borderline vascular tumors correlated to the results obtained in a diverse panel of malignant sarcoma cells, revealing immunoreactivity for Oct4, Nanog, and Sox2 in 100 % of various sarcoma tissues and 72 % for Myc and Kfl4 (Additional file 2: Table S2). While Klf4 protein expression was not significantly different between any of the vascular tumors or vascular tissue controls, this protein did show a significantly increased mean IHC score in the sarcoma tissue control set (Fig. 6, Additional file 2: Table S2). The expression of Oct4, Nanog, Myc, Sox2, and Klf4 in the current study correlated well with data reported in the HPA which reveals expression of Oct4 in 88 % of cancers, Sox2 in 88 % of cancers, Myc in 78 % of cancers, Klf4 in 28 % of cancers, and Nanog in 7 % of cancers.

## Discussion

This study directly stems from the results of a handful of recent publications which suggest that the benign vascular tumor, infantile hemangioma, harbors a subpopulation of stem cell-like progenitor cells. mRNA and protein expression of neural crest and stem cell markers was previously confirmed in a panel of hemangioma samples, revealing variable expression levels for Oct4, Myc, Sox2, and Nanog [10, 11]. These publications suggest that infantile hemangiomas may contain cells that are capable of differentiating into all three embryonic germ layers and additionally point to a possible mechanism of clonality in these tumors. Indeed, implantation of isolated CD133+ stem cell populations from infantile hemangiomas produce hemangioma-like tumors in xenograft animal models [14], however Oct4 and Nanog positive subpopulations from infantile hemangiomas failed to form teratomas in SCID/NOD mice [11], a hallmark of embryonic stem cell-derived tumors [15], suggesting they do not function like true embryonic stem cells. Substantial lines of evidence controversially suggest that congenital and infantile hemangiomas originate from metastatic spread of placental chorangiomas [16–20], creating a possibility in which the etiology of some childhood hemangiomas (at least in their earliest stages) may be more similar to metastatic tumors than benign tumors. Thus, our observations that both infantile hemangiomas and malignant vascular tumors such as angiosarcomas and hemangiopericytomas expressed stem cell reprogramming factors at significantly increased and relatively similar levels compared to non-diseased vascular tissue, is not entirely surprising.

In contrast, our highly novel observations that other benign vascular tumors such as adult capillary, cavernous, granulomatous, and venous hemangiomas as well as the single thrombophlebitis sample displayed expression of Oct4, Nanog, Myc, and Sox2 in similarly elevated rates and intensities as seen in malignant sarcomas was quite surprising. These benign tumors often occur in the third to fourth decade of life, thus their origin cannot be attributed easily to distal neoplasms as arguably may occur in infantile hemangiomas. These expression patterns in diverse benign vascular tumors are intriguing given that the presence of “stemness” proteins in malignant tumors is well established in the literature and forms the basis for the “stem cell theory of cancer”; however our data provide strong evidence that these proteins could potentially contribute to the formation and/or properties associated with a diverse array of benign vascular tumors. Though more studies must be performed for definitive arguments either way, it is possible that the “stem cell theory of cancer” is too narrowly defined in its current state and may need to be broadened to include benign neoplasms. This subject should be treaded lightly and with careful future evaluation as, while Oct4 has been shown to maintain pluripotency during early embryogenesis, its role as a pure stem cell marker has been questioned given that it is also expressed in differentiated cells [21, 22]. Nanog expression has been reported in E18 stage rat myocardial tissues, and is detectable in post-natal stages up to 30 days after birth and after acute myocardial infarction [23, 24].

Compared to the abundance of research performed in carcinomas and hematopoietic cancers, relatively minimal work has been reported evaluating the presence of stem cells as driving components in malignant mesenchymal tumors, and much of these efforts have focused exclusively on pediatric bone and musculoskeletal sarcomas [25–27]. For instance, osteosarcomas and Ewing’s sarcomas express Oct4 and Nanog [25, 28, 29] and rhabdomyosarcomas express Oct4, Nanog, and Sox2 [30]. Moreover, the EWS-FLI1 fusion gene, present in nearly 85 % of Ewing’s sarcomas, induces the expression of Oct4, Nanog, and Sox2 in human pediatric mesenchymal stem cells but not their adult counterparts [31]. Though drug resistant progenitor-like cell populations have been reported for angiosarcomas [32, 33], only expression of Myc as an embryonic stem cell marker has been thoroughly examined in malignant vascular tumors [34]. It has been reported that Myc gene amplification and overexpression occurs in post-irradiation induced angiosarcomas, but not in primary cutaneous angiosarcomas or in other radiation-associated vascular proliferations [35, 36]; however several other studies provide evidence that Myc amplification and overexpression is not a definitive marker of radiation-induced tumorigenesis in angiosarcomas [37–39]. Our data additionally demonstrates that Oct4, Nanog, Sox2, Klf4, and Myc are widely expressed at high levels across a wide variety of sarcomas and benign vascular tumors at elevated levels. While the data reported in this study in no way indicate that the cells expressing these markers are cancer stem cells (which generally make up single digit or less percentages of the total cancer cell population in a tumor), the statistically significant increases in Oct4, Nanog, Sox2, and Myc expression in benign and malignant tumors relative to normal tissues provides correlative support that overexpression of these proteins could contribute to their overall tumorigenic properties.

## Conclusion

In conclusion, the data presented in this study demonstrate that the protein expression of embryonic stem cell reprogramming factors is enriched in benign, borderline, and malignant vascular tumors. This finding could translate to future therapeutic targeting of tumor cell populations that express embryonic stem cell reprogramming factors to disrupt tumor cell clonality, long term growth, and drug resistance.

## Additional files

Additional file 1: Figure S1. Positive and negative staining controls. For each indicated antigen detected by immunohistochemistry, three controls were performed. The Negative Control column represents images acquired following immunohistochemistry against the indicated antigen on adipocyte tissue, which has been shown by the HPA to express no to low levels of each protein. The No Primary Antibody column represents images acquired following immunohistochemistry using no primary antibody on tissues as indicated in the Materials and Methods section to demonstrate that the detection system was no causing background staining on the samples. The Positive Control column represents images acquired from immunohistochemistry using the indicated antibody on tissues as indicated in the Materials and Methods section that are known to strongly express each antigen. (DOC 488 kb) Additional file 2: Table S1. Immunopositivity for stem cell reprogramming factors in malignant and benign vascular tumors. **Table S2.** Immunopositivity for stem cell reprogramming factors in a panel of diverse sarcomas. (DOC 214 kb)

## Abbreviations

CD133: Cluster of differentiation 133 protein
EWS-FLI1: Ewings sarcoma oncogene-friend leukemia virus integration 1
HPA: Human protein atlas
IHC: Immunohistochemistry
Klf4: Kruppel-like factor 4
Myc: Myelocytomatosis viral oncogene homolog
Nanog: Homeobox protein Nanog
Oct4: Octamer-binding transcription factor 3/4
SCID/NOD: Severe combined immunodeficiency disease/non-obese diabetic
SOX2: Sex determining region Y box 2 protein
TTUHSC: Texas Tech University Health Sciences Center
