# Supplementary material for: Enrichment of the embryonic stem cell reprogramming factors Oct4, Nanog, Myc, and Sox2 in benign and malignant vascular tumors
Source: BMC Clin Pathol. 2015 Sep 26;15:18. doi: 10.1186/s12907-015-0018-0 (PMC4584003; doi:10.1186/s12907-015-0018-0)
Supplement: Additional file 1: Figure S1. — Positive and negative staining controls. For each indicated antigen detected by immunohistochemistry, three controls were performed. The Negative Control column represents images acquired following immunohistochemistry against the indicated antigen on adipocyte tissue, which has been shown by the HPA to express no to low levels of each protein. The No Primary Antibody column represents images acquired following immunohistochemistry using no primary antibody on tissues as indicated in the Materials and Methods section to demonstrate that the detection system was no causing background staining on the samples. The Positive Control column represents images acquired from immunohistochemistry using the indicated antibody on tissues as indicated in the Materials and Methods section that are known to strongly express each antigen. (DOC 488 kb) [file 12907_2015_18_MOESM1_ESM.doc]

Additional Figure S1. Positive and negative staining controls. For each indicated antigen detected by immunohistochemistry, three controls were performed. The Negative Control column represents images acquired following immunohistochemistry against the indicated antigen on adipocyte tissue, which has been shown by the HPA to express no to low levels of each protein. The No Primary Antibody column represents images acquired following immunohistochemistry using no primary antibody on tissues as indicated in the Materials and Methods section to demonstrate that the detection system was no causing background staining on the samples. The Positive Control column represents images acquired from immunohistochemistry using the indicated antibody on tissues as indicated in the Materials and Methods section that are known to strongly express each antigen.
