# Supplementary material for: Enrichment of the embryonic stem cell reprogramming factors Oct4, Nanog, Myc, and Sox2 in benign and malignant vascular tumors
Source: BMC Clin Pathol. 2015 Sep 26;15:18. doi: 10.1186/s12907-015-0018-0 (PMC4584003; doi:10.1186/s12907-015-0018-0)
Supplement: Additional file 2: Table S1. — Immunopositivity for stem cell reprogramming factors in malignant and benign vascular tumors. Table S2. Immunopositivity for stem cell reprogramming factors in a panel of diverse sarcomas. (DOC 214 kb) [file 12907_2015_18_MOESM2_ESM.doc]

**Additional file 2 Table S1. Immunopositivity for stem cell reprogramming factors in malignant and benign vascular tumors.**

| **Location** | **Diagnosis** | **Type** |  | **Oct4** | **Nanog** | **Klf4** | **Sox2** | **Myc** |
| --- | --- | --- | --- | --- | --- | --- | --- | --- |
| Heart | Angiosarcoma | Malignant |  | ++ _>75%_ | + _50-75%_ | + _<25%_ | +++ _50-75%_ | + _<25%_ |
| Fibrous tissue | Angiosarcoma | Malignant |  | +++ _50-75%_ | ++ _<25%_ | + _<25%_ | + _25-50%_ | + _<25%_ |
| Liver | Angiosarcoma | Malignant |  | ++ _50-75%_ | ++ _<25%_ | ++ _<25%_ | ++ _50-75%_ | ++ _<25%_ |
| Blood vessel | Angiosarcoma | Malignant |  | +++ _25-50%_ | + _25-50%_ | + _<25%_ | ++ _<25%_ | + _<25%_ |
| Fallopian tube | Angiosarcoma | Malignant |  | + _50-75%_ | ++ _25-50%_ | + _<25%_ | + _<25%_ | 0 |
| Ankle | Angiosarcoma | Malignant |  | +++ _50-75%_ | + _<25%_ | 0 | ++ _50-75%_ | + _<25%_ |
| Spleen | Angiosarcoma | Malignant |  | ++ _>75%_ | ++ _<25%_ | 0 | ++ _<25%_ | 0 |
| Lung | Hemangiopericytoma | Malignant |  | ++ _>75%_ | + _25-50%_ | 0 | ++ _>75%_ | 0 |
| Skin | Hemangiopericytoma | Malignant |  | ++ _50-75%_ | + _25-50%_ | 0 | + _50-75%_ | 0 |
| Heart | Hemangioendothelioma | Borderline |  | +++ _50-75%_ | ++ _<25%_ | + _<25%_ | ++ _<25%_ | ++ _25-50%_ |
| Skin | Hemangioendothelioma | Borderline |  | ++ _50-75%_ | ++ _25-50%_ | 0 | + _<25%_ | + _<25%_ |
| Skin | Hemangioendothelioma | Borderline |  | ++ _50-75%_ | +++ _<25%_ | + _<25%_ | + _50-75%_ | + _<25%_ |
| Ligament | Hemangioendothelioma | Borderline |  | +++ _>75%_ | +++ _<25%_ | + _<25%_ | ++ _<25%_ | 0 |
| Blood vessel | Hemangioendothelioma | Borderline |  | +++ _>75%_ | +++ _25-50%_ | ++ _<25%_ | ++ _50-75%_ | 0 |
| Blood vessel | Hemangioendothelioma | Borderline |  | +++ _>75%_ | + _<25%_ | 0 | + _25-50%_ | 0 |
| Skin | Capillary hemangioma | Benign |  | +++ _50-75%_ | +++ _50-75%_ | ++ _<25%_ | ++ _25-50%_ | 0 |
| Skin | Capillary hemangioma | Benign |  | +++ _>75%_ | ++ _50-75%_ | + _<25%_ | ++ _<25%_ | + _<25%_ |
| Skin | Capillary hemangioma | Benign |  | +++ _50-75%_ | + _<25%_ | + _<25%_ | ++ _25-50%_ | + _<25%_ |
| Skin | Capillary hemangioma | Benign |  | +++ _50-75%_ | 0 | ++ _<25%_ | + _25-50%_ | + _<25%_ |
| Skin | Capillary hemangioma | Benign |  | ++ _50-75%_ | +++ _25-50%_ | ++ _<25%_ | +++ _25-50%_ | ++ _25-50%_ |
| Skin | Capillary hemangioma | Benign |  | +++ _50-75%_ | + _<25%_ | + _<25%_ | ++ _<25%_ | + _<25%_ |
| Liver | Cavernous hemangioma | Benign |  | +++ _50-75%_ | +++ _50-75%_ | ++ _<25%_ | +++ _25-50%_ | 0 |
| Liver | Cavernous hemangioma | Benign |  | +++ _>75%_ | + _<25%_ | + _<25%_ | + _<75%_ | + _<25%_ |
| Liver | Cavernous hemangioma | Benign |  | + _25-50%_ | ++ _<25%_ | 0 | + _<25%_ | + _<25%_ |
| Liver | Cavernous hemangioma | Benign |  | ++ _50-75%_ | + _<25%_ | + _<25%_ | + _25-50%_ | + _<25%_ |
| Liver | Cavernous hemangioma | Benign |  | ++ _50-75%_ | + _<25%_ | + _<25%_ | + _25-50%_ | ++ _<25%_ |
| Liver | Cavernous hemangioma | Benign |  | ++ _50-75%_ | + _<25%_ | + _<25%_ | + _<25%_ | 0 |
| Liver | Cavernous hemangioma | Benign |  | ++ _50-75%_ | ++ _<25%_ | + _<25%_ | + _<25%_ | 0 |
| Liver | Cavernous hemangioma | Benign |  | ++ _50-75%_ | +++ _<25%_ | 0 | ++ _25-50%_ | + _<25%_ |
| Cerebrum | Cavernous hemangioma | Benign |  | ++ _50-75%_ | ++ _25-50%_ | ++ _<25%_ | ++ _25-50%_ | + _<25%_ |
| Liver | Cavernous hemangioma | Benign |  | ++ _25-50%_ | +++ _50-75%_ | ++ _<25%_ | 0 | 0 |
| Liver | Cavernous hemangioma | Benign |  | ++ _50-75%_ | ++ _25-50%_ | ++ _<25%_ | + _25-50%_ | + _<25%_ |
| Liver | Cavernous hemangioma | Benign |  | + _25-50%_ | + _25-50%_ | + _<25%_ | + _<25%_ | 0 |
| Liver | Cavernous hemangioma | Benign |  | ++ _25-50%_ | + _<25%_ | 0 | + _<25%_ | 0 |
| Liver | Cavernous hemangioma | Benign |  | ++ _25-50%_ | ++ _<25%_ | 0 | + _<25%_ | 0 |
| Liver | Cavernous hemangioma | Benign |  | ++ _50-75%_ | ++ _25-50%_ | + _<25%_ | ++ _<25%_ | + _<25%_ |
| Liver | Cavernous hemangioma | Benign |  | +++ _50-75%_ | ++ _25-50%_ | + _<25%_ | ++ _25-50%_ | 0 |
| Liver | Cavernous hemangioma | Benign |  | ++ _<25%_ | ++ _25-50%_ | + _<25%_ | + _25-50%_ | 0 |
| Liver | Cavernous hemangioma | Benign |  | ++ _25-50%_ | + _<25%_ | 0 | + _25-50%_ | 0 |
| Liver | Cavernous hemangioma | Benign |  | ++ _50-75%_ | +++ _<25%_ | 0 | + _<25%_ | 0 |
| Liver | Cavernous hemangioma | Benign |  | ++ _50-75%_ | ++ _<25%_ | 0 | ++ _<25%_ | + _<25%_ |
| Liver | Cavernous hemangioma | Benign |  | ++ _<25%_ | +++ _50-75%_ | 0 | ++ _<25%_ | 0 |
| Liver | Cavernous hemangioma | Benign |  | +++ _50-75%_ | + _25-50%_ | + _<25%_ | + _<25%_ | 0 |
| Liver | Cavernous hemangioma | Benign |  | +++ _50-75%_ | +++ _<25%_ | ++ _<25%_ | + _<25%_ | 0 |
| Liver | Cavernous hemangioma | Benign |  | + _25-50%_ | + _25-50%_ | 0 | + _<25%_ | 0 |
| Liver | Cavernous hemangioma | Benign |  | +++ _50-75%_ | ++ _25-50%_ | 0 | + _<25%_ | 0 |
| Liver | Cavernous hemangioma | Benign |  | +++ _50-75%_ | + _25-50%_ | 0 | + _25-50%_ | 0 |
| Liver | Cavernous hemangioma | Benign |  | +++ _50-75%_ | ++ _<25%_ | 0 | + _25-50%_ | + _<25%_ |
| Liver | Cavernous hemangioma | Benign |  | + _25-50%_ | ++ _<25%_ | 0 | + _<25%_ | 0 |
| Liver | Cavernous hemangioma | Benign |  | ++ _25-50%_ | + _50-75%_ | 0 | + _<25%_ | + _<25%_ |
| Liver | Cavernous hemangioma | Benign |  | ++ _50-75%_ | ++ _<25%_ | 0 | + _<25%_ | 0 |
| Liver | Cavernous hemangioma | Benign |  | ++ _50-75%_ | ++ _25-50%_ | 0 | ++ _25-50%_ | 0 |
| Liver | Cavernous hemangioma | Benign |  | ++ _25-50%_ | +++ _<25%_ | 0 | + _<25%_ | 0 |
| Liver | Cavernous hemangioma | Benign |  | +++ _25-50%_ | ++ _<25%_ | 0 | ++ _<25%_ | + _<25%_ |
| Liver | Cavernous hemangioma | Benign |  | +++ _25-50%_ | +++ _50-75%_ | + _<25%_ | + _<25%_ | 0 |
| Liver | Cavernous hemangioma | Benign |  | +++ _25-50%_ | ++ _<25%_ | + _<25%_ | + _<25%_ | 0 |
| Liver | Cavernous hemangioma | Benign |  | +++ _50-75%_ | + _<25%_ | + _<25%_ | + _50-75%_ | 0 |
| Cerebrum | Cavernous hemangioma | Benign |  | +++ _50-75%_ | +++ _25-50%_ | +++ _<25%_ | +++ _50-75%_ | +++ _<25%_ |
| Spleen | Cavernous hemangioma | Benign |  | ++ _50-75%_ | +++ _50-75%_ | 0 | ++ _25-50%_ | + _<25%_ |
| Tongue | Cavernous hemangioma | Benign |  | +++ _50-75%_ | +++ _25-50%_ | + _<25%_ | +++ _50-75%_ | + I |
| Thyroid | Cavernous hemangioma | Benign |  | ++ _<25%_ | ++ _25-50%_ | 0 | + _<25%_ | 0 |
| Skin | Cavernous hemangioma | Benign |  | +++ _50-75%_ | ++ _25-50%_ | + _<25%_ | ++ _25-50%_ | ++ _25-50%_ |
| Vulva | Cavernous hemangioma | Benign |  | ++ _25-50%_ | ++ _25-50%_ | + _<25%_ | ++ _<25%_ | 0 |
| Blood vessel | Cavernous hemangioma | Benign |  | ++ _50-75%_ | ++ _50-75%_ | ++ _<25%_ | ++ _<25%_ | + _<25%_ |
| Spleen | Cavernous hemangioma | Benign |  | +++ _25-50%_ | ++ _50-75%_ | 0 | ++ _<25%_ | 0 |
| Spleen | Cavernous hemangioma | Benign |  | +++ _25-50%_ | +++ _25-50%_ | + _<25%_ | ++ _25-50%_ | 0 |
| Tongue | Granulomatous hemangioma | Benign |  | ++ _50-75%_ | +++ _50-75%_ | 0 | ++ _50-75%_ | + _<25%_ |
| Skin | Granulomatous hemangioma | Benign |  | +++ _25-50%_ | + _<25%_ | ++ _25-50%_ | ++ _25-50%_ | ++ _25-50%_ |
| Skin | Granulomatous hemangioma | Benign |  | +++ _50-75%_ | +++ _50-75%_ | 0 | + _50-75%_ | ++ _<25%_ |
| Blood vessel | Phlebitis with thrombus | Benign |  | ++ _25-50%_ | ++ _50-75%_ | + _<25%_ | ++ _25-50%_ | 0 |
| Mesentery | Venous hemangioma | Benign |  | +++ _>75%_ | ++ _<25%_ | + _<25%_ | + _<25%_ | + _<25%_ |
| Carotid artery | Non-diseased | Normal |  | 0 | 0 | 0 | 0 | 0 |
| Carotid artery | Non-diseased | Normal |  | ++ _25-50%_ | 0 | 0 | 0 | 0 |
| Carotid artery | Non-diseased | Normal |  | + _<25%_ | 0 | 0 | 0 | 0 |
| Carotid artery | Non-diseased | Normal |  | + _25-50%_ | + _<25%_ | 0 | + _<25%_ | 0 |
| Carotid artery | Non-diseased | Normal |  | ++ _25-50%_ | ++ _<25%_ | ++ _<25%_ | + _<25%_ | 0 |
| Aorta | Non-diseased | Normal |  | ++ _25-50%_ | ++ _<25%_ | + _25-50%_ | ++ _<25%_ | 0 |
| Aorta | Non-diseased | Normal |  | + _<25%_ | 0 | 0 | + _<25%_ | 0 |
| Aorta | Non-diseased | Normal |  | ++ _25-50%_ | + _<25%_ | + _50-75%_ | + _<25%_ | 0 |
| Aorta | Non-diseased | Normal |  | + _25-50%_ | ++ _<25%_ | + _25-50%_ | + _25-50%_ | 0 |
| Aorta | Non-diseased | Normal |  | ++ _<25%_ | 0 | + _<25%_ | 0 | 0 |

0 = no expression; + = low expression; ++ = moderate expression; +++ = high expression; % = amount of positivity in tissue.

**Additional file 2 Table S2. Immunopositivity for stem cell reprogramming factors in a panel of diverse sarcomas.**

| **Location** | **Diagnosis** | **Oct4** | **Nanog** | **Klf4** | **Sox2** | **Myc** |
| --- | --- | --- | --- | --- | --- | --- |
| Coccyx | Chordoma | +++ _50-75%_ | ++ _<25%_ | ++ _<25%_ | ++ _50-75%_ | ++ _50-75%_ |
| Coccyx | Chordoma | +++ _50-75%_ | ++ _<25%_ | ++ _<25%_ | ++ _>75%_ | + _<25%_ |
| Sacrum | Chordoma | ++ _25-50%_ | ++ _50-75%_ | ++ _25-50%_ | ++ _25-50%_ | + _<25%_ |
| Soft tissue, face | Dermatofibrosarcoma protuberans | +++ _50-75%_ | ++ _25-50%_ | +++ _<25%_ | + _25-50%_ | + _<25%_ |
| Small bowel | Desmoplastic small round cell tumor | +++ _50-75%_ | + _<25%_ | + _<25%_ | +++ _50-75%_ | + _25-50%_ |
| Soft tissue, thigh | Epithelioid sarcoma | +++ _50-75%_ | + _<25%_ | ++ _50-75%_ | ++ _50-75%_ | + _<25%_ |
| Soft tissue, thigh | Fibrosarcoma | +++ _>75%_ | + _<25%_ | + _<25%_ | ++ _>75%_ | 0 |
| Kidney | Leiomyosarcoma | ++ _>75%_ | 0 | + _<25%_ | + _50-75%_ | 0 |
| Uterus | Leiomyosarcoma | +++ _>75%_ | ++ _25-50%_ | ++ _50-75%_ | ++ _>75%_ | ++ _50-75%_ |
| Abdominal cavity | Leiomyosarcoma | +++ _>75%_ | + _25-50%_ | ++ _>75%_ | + _25-50%_ | + _25-50%_ |
| Pelvic cavity | Leiomyosarcoma | ++ _>75%_ | ++ _<25%_ | ++ _50-75%_ | + _50-75%_ | 0 |
| Soft tissue, thigh | Leiomyosarcoma | +++ _>75%_ | ++ _<25%_ | + _<25%_ | +++ _>75%_ | + _50-75%_ |
| Soft tissue, thigh | Leiomyosarcoma | ++ _50-75%_ | + _25-50%_ | + _<25%_ | ++ _50-75%_ | 0 |
| Spermatic cord | Leiomyosarcoma | +++ _50-75%_ | +++ _50-75%_ | + _25-50%_ | +++ _50-75%_ | ++ _25-50%_ |
| Soft tissue, chest | Leiomyosarcoma | +++ _50-75%_ | + _<25%_ | ++ _50-75%_ | + _>75%_ | 0 |
| Retroperioneum | Leiomyosarcoma | ++ _50-75%_ | + _<25%_ | + _<25%_ | +++ _>75%_ | 0 |
| Retroperioneum | Liposarcoma | +++ _50-75%_ | +++ _25-50%_ | + _50-75%_ | + _50-75%_ | + _<25%_ |
| Retroperioneum | Liposarcoma | ++ _>75%_ | + _<25%_ | + _<25%_ | + _>75%_ | 0 |
| Abdominal cavity | Liposarcoma | +++ _50-75%_ | + _<25%_ | ++ _<25%_ | ++ _<25%_ | + _<25%_ |
| Mesentery | Liposarcoma | +++ _50-75%_ | ++ _25-50%_ | ++ _<25%_ | +++ _<25%_ | ++ _<25%_ |
| Soft tissue, axilla | Liposarcoma | ++ _25-50%_ | ++ _<25%_ | 0 | ++ _<25%_ | + _<25%_ |
| Soft tissue, lower leg | Liposarcoma | ++ _50-75%_ | ++ _25-50%_ | + _<25%_ | ++ _<25%_ | ++ _25-50%_ |
| Soft tissue, thigh | Lipsarcoma | ++ _50-75%_ | ++ _25-50%_ | + _<25%_ | + _50-75%_ | + _25-50%_ |
| Femur | Malignant giant cell tumor | +++ _50-75%_ | +++ _>75%_ | + _25-50%_ | +++ _50-75%_ | + _25-50%_ |
| Soft tissue, hip | Mesenchymoma, malignant | +++ _>75%_ | ++ _<25%_ | ++ _<25%_ | ++ _50-75%_ | ++ _<25%_ |
| Descending colon | Neurofibrosarcoma | +++ _>75%_ | ++ _25-50%_ | 0 | +++ _50-75%_ | 0 |
| Soft tissue, forearm | Neurofibrosarcoma | +++ _>75%_ | + _<25%_ | ++ _25-50%_ | + _50-75%_ | + _<25%_ |
| Soft tissue, back | Osteosarcoma | +++ _>75%_ | +++ _<25%_ | +++ _<25%_ | ++ _50-75%_ | + _<25%_ |
| Soft tissue, thigh | Osteosarcoma | +++ _>75%_ | +++ _<25%_ | + _50-75%_ | + _>75%_ | + _25-50%_ |
| Pelvic cavity | Perivascular epithelioid neoplasm | ++ _50-75%_ | ++ _25-50%_ | ++ _<25%_ | ++ _50-75%_ | ++ _<25%_ |
| Soft tissue, knee | Pleomorphic rhabdomyosarcoma | +++ _>75%_ | ++ _50-75%_ | + _25-50%_ | +++ _50-75%_ | ++ _25-50%_ |
| Soft tissue, thigh | Pleomorphic undifferentiated sarcoma | +++ _50-75%_ | ++ _25-50%_ | ++ _<25%_ | +++ _50-75%_ | 0 |
| Soft tissue, vertebra | Pleomorphic undifferentiated sarcoma | +++ _>75%_ | + _<25%_ | + _25-50%_ | ++ _50-75%_ | + _25-50%_ |
| Soft tissue, knee | Pleomorphic undifferentiated sarcoma | ++ _50-75%_ | + _<25%_ | ++ _<25%_ | ++ _50-75%_ | 0 |
| Soft tissue, upper arm | Pleomorphic undifferentiated sarcoma | +++ _>75%_ | ++ _<25%_ | ++ _<25%_ | + _>75%_ | + _25-50%_ |
| Soft tissue, neck | Pleomorphic undifferentiated sarcoma | +++ _>75%_ | ++ _50-75%_ | +++ _<25%_ | ++ _>75%_ | ++ _25-50%_ |
| Soft tissue, chest | Pleomorphic undifferentiated sarcoma | ++ _50-75%_ | ++ _25-50%_ | + _<25%_ | ++ _50-75%_ | 0 |
| Soft tissue, neck | Pleomorphic undifferentiated sarcoma | +++ _>75%_ | ++ _25-50%_ | ++ _50-75%_ | +++ _>75%_ | + _25-50%_ |
| Soft tissue, knee | Pleomorphic undifferentiated sarcoma | +++ _>75%_ | ++ _50-75%_ | ++ _50-75%_ | +++ _>75%_ | ++ _25-50%_ |
| Soft tissue, knee | Pleomorphic undifferentiated sarcoma | +++ _>75%_ | + _<25%_ | + _25-50%_ | +++ _50-75%_ | + _<25%_ |
| Soft tissue, knee | Pleomorphic undifferentiated sarcoma | ++ _50-75%_ | + _25-50%_ | + _<25%_ | ++ _50-75%_ | 0 |
| Soft tissue and femur, thigh | Pleomorphic undifferentiated sarcoma | +++ _>75%_ | ++ _<25%_ | + _<25%_ | ++ _50-75%_ | + _25-50%_ |
| Soft tissue, thigh | Pleomorphic undifferentiated sarcoma | +++ _50-75%_ | + _>75%_ | + _50-75%_ | +++ _50-75%_ | + _50-75%_ |
| Abdominal cavity | Primitive neuroectodermal tumor | +++ _50-75%_ | +++ _<25%_ | + _<25%_ | +++ _>75%_ | + _<25%_ |
| Soft tissue, pelvis | Primitive neuroectodermal tumor | +++ _>75%_ | + _50-75%_ | + _<25%_ | +++ _25-50%_ | ++ _<25%_ |
| Pelvic cavity | Primitive neuroectodermal tumor | +++ _>75%_ | + _<25%_ | + _25-50%_ | +++ _>75%_ | + _25-50%_ |
| Kidney | Primitive neuroectodermal tumor | +++ _50-75%_ | ++ _<25%_ | + _25-50%_ | ++ _50-75%_ | 0 |
| Soft tissue, cheek | Rhabdomyosarcoma | +++ _50-75%_ | ++ _<25%_ | ++ _<25%_ | +++ _>75%_ | + _<25%_ |
| Retroperioneum | Rhabdomyosarcoma | ++ _50-75%_ | +++ _25-50%_ | + _<25%_ | + _50-75%_ | 0 |
| Soft tissue, arm | Rhabdomyosarcoma | +++ _>75%_ | ++ _<25%_ | + _<25%_ | + _50-75%_ | 0 |
| Soft tissue, shoulder | Synovial sarcoma | ++ _50-75%_ | +++ _25-50%_ | + _<25%_ | ++ _>75%_ | + _25-50%_ |
| Abdominal wall | Synovial sarcoma | +++ _>75%_ | ++ _50-75%_ | + _25-50%_ | +++ _>75%_ | + _50-75%_ |
| Soft tissue, thigh | Synovial sarcoma | ++ _>75%_ | + _<25%_ | + _25-50%_ | ++ _>75%_ | ++ _25-50%_ |
| Soft tissue, thigh | Synovial sarcoma | +++ _>75%_ | + _25-50%_ | + _25-50%_ | +++ _50-75%_ | ++ _25-50%_ |
| Soft tissue, thigh | Synovial sarcoma | +++ _50-75%_ | + _50-75%_ | + _25-50%_ | ++ _50-75%_ | + _25-50%_ |
| Soft tissue, chest wall | Synovial sarcoma | +++ _>75%_ | ++ _<25%_ | ++ _<25%_ | ++ _>75%_ | + _<25%_ |
| Soft tissue, pelvis | Synovial sarcoma | +++ _>75%_ | + _<25%_ | + _<25%_ | ++ _>75%_ | 0 |
| Soft tissue, thigh | Synovial sarcoma | +++ _>75%_ | +++ _<25%_ | + _25-50%_ | +++ _50-75%_ | + _50-75%_ |

0 = no expression; + = low expression; ++ = moderate expression; +++ = high expression; % = amount of positivity in tissue.
